# Study on the Structure, Thermal Properties and Antibacterial Properties of Phosphorus-Modified PVA/TiO_2_ Composite Films

**DOI:** 10.3390/gels11121020

**Published:** 2025-12-18

**Authors:** Alina-Mirela Ipate, Diana Serbezeanu, Ioana-Antonia Iftimie, Gabriela Lisa, Cristina-Mihaela Rîmbu, Tăchiță Vlad-Bubulac

**Affiliations:** 1 “Petru Poni” Institute of Macromolecular Chemistry, Aleea Gr. Ghica Voda 41A, 700487 Iasi, Romania; diana.serbezeanu@icmpp.ro (D.S.); iftimie.ioana@icmpp.ro (I.-A.I.); 2 Department of Chemical Engineering, Faculty of Chemical Engineering and Environmental Protection, Gheorghe Asachi Technical University of Iasi, Bd. Mangeron 73, 700050 Iasi, Romania; gabriela.lisa@academic.tuiasi.ro; 3 Department of Public Health, Faculty of Veterinary Medicine “Ion Ionescu de la Brad”, University of Agricultural Sciences and Veterinary Medicine, 8, Mihail Sadoveanu Alley, 707027 Iasi, Romania; cristina.rimbu@iuls.ro

**Keywords:** phosphorylated PVA, TiO_2_ NPs, hybrid composites, thermal stability, antimicrobial activity

## Abstract

Phosphorus-modified poly(vinyl alcohol) (PVA) has recently gained increasing attention as a functional polymeric matrix suitable for gel-based systems, owing to its biocompatibility, film-forming ability, and capacity to develop semi-interpenetrating networks. In this work, PVA was chemically modified through the nucleophilic substitution of its hydroxyl groups with the chloride groups of phenyl dichlorophosphate, following a literature-reported method carried out in N,N-dimethylformamide (DMF) as reaction medium, resulting in phosphorus-containing PVA networks (PVA-OP3). Hybrid gel-like films were then prepared by incorporating titanium dioxide nanoparticles (TiO_2_ NPs), known for their antimicrobial activity, low toxicity, and high stability. The resulting composites were structurally, morphologically, and thermally characterized using FTIR, SEM, and thermogravimetric analysis. The incorporation of TiO_2_ NPs significantly improved the thermal stability, with T_5_% increasing from 240 °C for neat PVA-OP3 to 288 °C for the optimal composite, increased the char residue from 4.5% for the neat polymer to 30.1% for PVA-OP3/TiO_2_-4, and enhanced antimicrobial activity against both Gram-positive and Gram-negative bacteria. These findings demonstrate that PVA-OP3/TiO_2_ hybrid films possess promising potential as advanced biomaterials for biomedical, protective, and environmental applications.

## 1. Introduction

The development of advanced biomaterials has received increasing attention in recent decades, driven by the demand for multifunctional systems that combine structural integrity with enhanced biological performance [1,2,3,4]. Among the wide range of polymeric materials, polyvinyl alcohol (PVA) has gained considerable attention due to its attractive combination of physicochemical and biological properties, which originate from its highly hydrophilic backbone rich in hydroxyl groups capable of extensive hydrogen bonding. This molecular architecture provides PVA with excellent film-forming ability, mechanical flexibility, and tunable crystallinity, enabling precise control over its structural organization and performance in functional materials. In addition, PVA demonstrates notable biocompatibility, low cytotoxicity, and biodegradability under physiological or enzyme-mediated conditions, which make it suitable for a broad spectrum of biomedical applications, including drug delivery, tissue engineering scaffolds, wound dressings, and bioinks for 3D printing. From a processing standpoint, PVA is readily shaped into hydrogels, nanofibers, thin films, and composite structures due to its solubility in water and compatibility with various crosslinking strategies – ranging from physical crosslinking through freeze–thaw cycles to chemical crosslinking using aldehydes, borates, phosphonic dichloride, or photoactive agents. These characteristics enable control over swelling behavior, mechanical robustness, degradation rate, and permeability, allowing application-specific customization [5,6,7,8,9,10,11].

Furthermore, the abundance of hydroxyl groups offers numerous sites for chemical modification and functionalization. Incorporation of phosphorus-containing functionalities enhances PVA’s chemical reactivity and imparts additional features such as improved thermal stability, flame retardancy, ionic interactions, and increased affinity toward metallic or ceramic nanoparticles [12,13,14]. Such modifications expand the applicability of PVA into fields requiring enhanced performance, including regenerative medicine, advanced coatings, and hybrid nanocomposites. Overall, the intrinsic versatility, biocompatibility, and chemical tunability of PVA position it as a valuable platform for designing next-generation multifunctional materials [12].

Titanium dioxide nanoparticles (TiO_2_ NPs) are another class of materials widely investigated for biomedical and environmental applications [15,16,17,18]. Their unique features, including low cytotoxicity, chemical stability, photocatalytic activity, and broad-spectrum antimicrobial effects, render them highly versatile [17,19,20]. The incorporation of TiO_2_ into polymeric matrices not only improves the mechanical and thermal behavior of the composites but also imparts functional properties that are valuable for protective coatings, wound dressings, and antimicrobial films [15,20,21,22,23,24,25,26,27]. A large number of studies have reported the incorporation of TiO_2_ into polymeric matrices, particularly into PVA, in order to enhance the overall performance of the resulting composites [21,22,24,26,28,29]. The interaction between the polymer chains and nanoparticles facilitates a uniform distribution of the inorganic phase and often leads to intermolecular bonding (hydrogen or electrostatic interactions), thereby reinforcing the structural integrity of the composite [30,31,32].

According to the literature, the combination of PVA with TiO_2_ has shown several advantages, including improved thermal and mechanical stability, reduced permeability to gases and water vapor, as well as the addition of functional properties such as UV resistance and antibacterial activity [21,22,28,33,34,35,36]. The improved thermal performance has been widely reported and is generally attributed to the presence of TiO_2_ nanoparticles, which enhance the structural integrity of the polymer matrix and contribute to more efficient thermal barrier effects [37]. The integration of TiO_2_ into PVA modified with phosphorus can further enhance these effects, as phosphorus groups in the polymer backbone promote stronger compatibility and more uniform dispersion of the nanoparticles. This synergy not only stabilizes the material but also amplifies its antimicrobial and protective functions.

Therefore, by combining PVA modified with phosphorus with TiO_2_ nanoparticles, it becomes possible to design hybrid films that leverage the complementary advantages of both components. The innovative aspect of this work lies in the chemical phosphorylation of PVA, which induces the formation of semi-interpenetrating networks through phosphate bridges. These networks enhance the structural integrity and thermal stability of the polymer matrix compared with unmodified PVA or conventional PVA/TiO_2_ composites. Incorporation of TiO_2_ nanoparticles into the phosphorus-modified PVA matrix further combines these stability improvements with antimicrobial functionality. This strategy provides a versatile and multifunctional platform for hybrid gel-like films, offering clear advantages for biomedical and protective applications over previously reported systems.

## 2. Results and Discussion

Semi-interpenetrated phosphorylated PVA-OP3/TiO_2_-0 film and the binary PVA-OP3/TiO_2_-1 to PVA-OP3/TiO_2_-5 nanocomposite gel-like films have been obtained by the casting from solution procedure. The details of this procedure are presented in the Section 4. The polymer matrix used as the starting material in this study, PVA-OP3, was prepared in our laboratory following the procedure described elsewhere [12]. The composition of all polymer matrices expressed in mass ratio for all the ingredients utilized in the preparation and the codes of the as-prepared composites are listed in Table 1.

### 2.1. Morphological and Elemental Analysis of the PVA-OP3/TiO_2_ Composites

**Figure 1:**
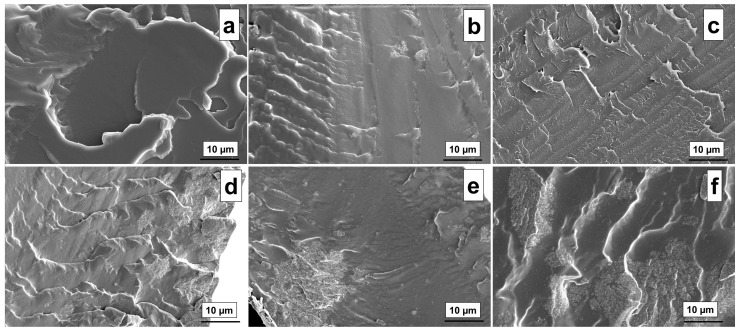
SEM images of the investigated samples PVA-OP3/TiO_2_-0 (**a**), PVA-OP3/TiO_2_-1 (**b**), PVA-OP3/TiO_2_-2 (**c**), PVA-OP3/TiO_2_-3 (**d**), PVA-OP3/TiO_2_-4 (**e**), PVA-OP3/TiO_2_-5 (**f**).

The surface morphology of the PVA-OP3/TiO_2_ nanocomposites was examined using SEM to investigate the effect of TiO_2_ loading on the microstructural features of the films (Figure 1a–f). The neat PVA-OP3 sample (Figure 1a) exhibited a relatively smooth and homogeneous fracture surface, characteristic of a continuous polymeric matrix without inorganic fillers. Upon the incorporation of a small amount of TiO_2_ (Figure 1b), slight surface roughness and fine striations appeared, suggesting good dispersion of the nanoparticles and strong interfacial interactions between TiO_2_ and the polymer chains [38]. As the TiO_2_ content increased (Figure 1c,d), the surfaces became progressively rougher, displaying localized irregularities and microcracks indicative of partial nanoparticle agglomeration and reduced matrix continuity [39]. At higher TiO_2_ concentrations (Figure 1e,f), the fracture surfaces displayed pronounced particle clusters and more irregular fractured regions. These features imply that, at higher loadings, the polymer matrix could no longer ensure effective nanoparticle dispersion. Due to the intrinsically high surface energy of TiO_2_, elevated filler contents promote particle–particle interactions over particle-polymer interactions, leading to the formation of agglomerated domains. Such agglomerates can be responsible for disrupting the structural uniformity of the matrix and hindering the development of strong interfacial adhesion, ultimately resulting in discontinuous regions and heterogeneous surface morphologies [40,41].

**Figure 2:**
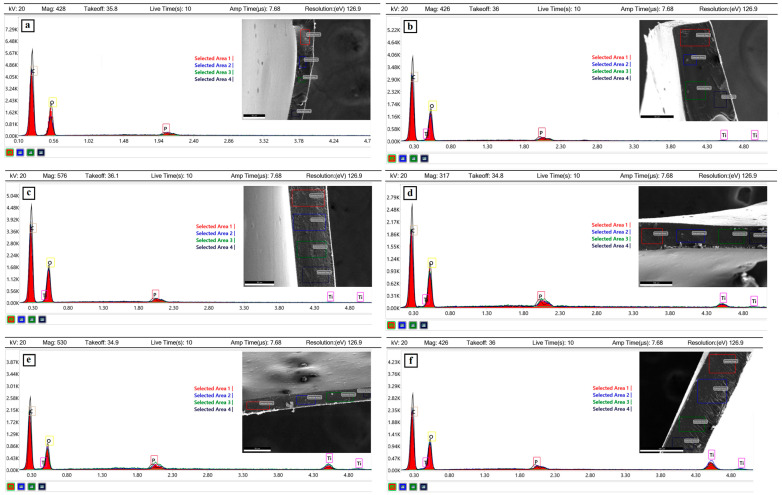
EDX spectra for the investigated samples (**a**) PVA-OP3/TiO_2_-0, (**b**) PVA-OP3/TiO_2_-1, (**c**) PVA-OP3/TiO_2_-2, (**d**) PVA-OP3/TiO_2_-3, (**e**) PVA-OP3/TiO_2_-4, (**f**) PVA-OP3/TiO_2_-5.

The incorporation of TiO_2_ within the prepared samples was further confirmed through Energy Dispersive X-ray Spectroscopy (EDX) analysis (Figure 2). The EDX spectra clearly exhibit characteristic peaks corresponding to titanium (Ti) in the energy range of approximately 4–5 keV, which are consistent with the reported values for Ti Kα and Ti Kβ emissions (4.508 keV and 4.93 keV, respectively) [42]. The presence of these peaks verifies the successful introduction of TiO_2_ into the PVA-OP3 matrix. In addition, the relative intensity of the Ti peaks in the different composite samples provides a qualitative indication of the TiO_2_ loading, thereby supporting the compositional variations among the PVA-OP3/TiO_2_-1 to PVA-OP3/TiO_2_-5 films (Figure 2b–f).

**Figure 3:**
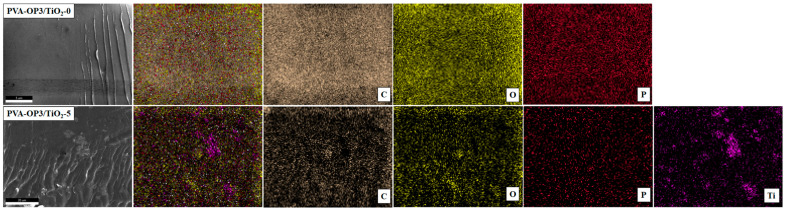
SEM micrographs and corresponding EDS elemental mapping images of PVA-OP3/TiO_2_ composites: (**top** row) PVA-OP3/TiO_2_-0 and (**bottom** row) PVA-OP3/TiO_2_-5.

The elemental distribution of the PVA-OP3/TiO_2_-0 and PVA-OP3/TiO_2_-5 composites was examined through SEM–EDS mapping to confirm the successful incorporation and dispersion of TiO_2_ nanoparticles within the polymer matrix (Figure 3). For the PVA-OP3/TiO_2_-0 sample, the elements C, O, and P—originating from the PVA-OP3 matrix—were homogeneously distributed across the analyzed area. In contrast, the PVA-OP3/TiO_2_-5 sample exhibited heterogeneous Ti distribution, with distinct regions of Ti enrichment suggesting nanoparticle agglomeration at higher loadings. The elemental mapping thus corroborates the SEM observations, revealing that excessive TiO_2_ content promotes particle clustering and phase separation within the composite structure.

For the PVA-OP3/TiO_2_-0 sample, the elements C, O, and P—originating from the PVA-OP3 matrix—were homogeneously distributed across the analyzed area. In contrast, the PVA-OP3/TiO_2_-5 sample exhibited heterogeneous Ti distribution, with distinct regions of Ti enrichment suggesting nanoparticle agglomeration at higher loadings. The elemental mapping thus corroborates the SEM observations, revealing that excessive TiO_2_ content promotes particle clustering and phase separation within the composite structure.

### 2.2. Structural Characterization

**Figure 4:**
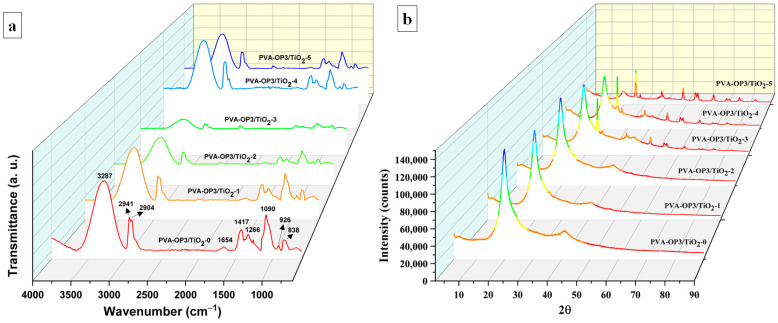
FTIR spectrum (**a**) of and XRD curves of the PVA-OP3/TiO_2_ composites (**b**).

FTIR and XRD provide complementary insights into the incorporation of TiO_2_ within the PVA-OP3 matrix, with FTIR revealing polymer–TiO_2_ interactions at the molecular level and XRD confirming the presence of the anatase TiO_2_ phase with retained crystalline structure. The FTIR spectra (Figure 4a) of the investigated samples display the characteristic bands of PVA, along with clear signatures associated with the phosphorus-containing groups and the incorporated TiO_2_. All spectra display the broad O–H stretching band of PVA centered in the 3200–3600 cm^−1^ region and the C–H stretching bands near 2900–2950 cm^−1^; the typical C–O/C–O–C vibrations of the PVA backbone appear in the 1080–1140 cm^−1^ region. The phosphorus modification gives rise to additional bands in the 1250–900 cm^−1^ region (assigned to P=O and P–O–C/P–O–P vibrations) [12,13,14,43], which are present in PVA-OP3/TiO_2_-0 and remain in the composites but change in shape and intensity upon TiO_2_ addition. Upon TiO_2_ incorporation, these bands exhibit subtle shifts, changes in intensity, and band broadening, indicating modifications of the local chemical environment. In addition, new absorptions emerge in the 400–700 cm^−1^ region, attributable to Ti–O–Ti and Ti–O stretching modes [35,36]. The observed spectral changes, including (i) shifts and broadening of the O–H and P-related bands and (ii) growth of Ti–O features, are consistent with specific polymer–nanoparticle interactions. These include hydrogen bonding between PVA hydroxyl groups and TiO_2_ surface sites, as well as possible coordination of phosphorus-containing groups to titanium atoms, potentially leading to the formation of the Ti–O–P linkages. Such interactions reduce the “free” character of O–H and P=O groups and contribute to the characteristic Ti–O vibrational envelope [35,36,44,45]. Overall, the FTIR data support successful incorporation of TiO_2_ into the PVA-OP3 matrix and indicate increasing polymer–oxide interaction as the TiO_2_ content increases.

The X-ray diffractograms (Figure 4b) show a broad polymeric halo centered near 18–20° 2θ for the TiO_2_-free sample (PVA-OP3/TiO_2_-0), consistent with the semi-crystalline nature of PVA. Upon incorporation of TiO_2,_ sharp reflections emerge at ≈25.3°, 37.8°, 48.0°, 53.9°, and 62.7°, which correspond to the (101), (004), (200), (105)/(211), and (204) planes of anatase TiO_2_, respectively. The intensity of these peaks increases progressively with TiO_2_ loading (from 0.0011 to 0.0388), indicating a higher proportion of crystalline TiO_2_ in the composite and good preservation of the anatase phase. No additional crystalline phases or significant peak shifts were detected, suggesting that TiO_2_ remains structurally intact and that interactions with the PVA-OP3 matrix are primarily interfacial rather than substitutional.

### 2.3. Thermal Stability of the PVA-OP3/TiO_2_ Composites

**Figure 5:**
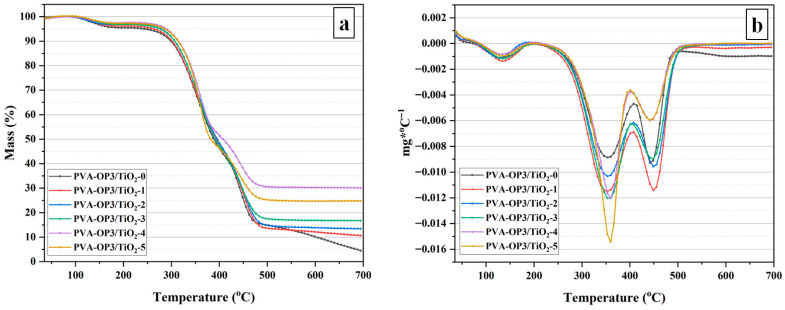
TG (**a**) and DTG curves (**b**) of the investigated composites.

The thermal properties of the PVA-OP3/TiO_2_ composites were evaluated using DSC, TGA, and DTG (Figure 5a,b), and the key parameters are summarized in Table 2. The glass transition temperature (*T_g_*) of neat PVA-OP3/TiO_2_-0 was 75.33 °C, and a slight increase was observed upon TiO_2_ incorporation, reaching a maximum of 79.15 °C for PVA-OP3/TiO_2_-1. This shift indicates restricted segmental mobility of the polymer chains due to interfacial interactions, likely hydrogen bonding, between TiO_2_ nanoparticles and the PVA-OP3 matrix. At higher TiO_2_ loadings, *T_g_* values fluctuated slightly but remained above that of the pure polymer, suggesting that the filler maintains a moderate reinforcing effect on chain rigidity. Thermogravimetric parameters (T_5%_, T_30%_, and T_HRI_) reveal a consistent enhancement in thermal stability with increasing TiO_2_ content up to PVA-OP3/TiO_2_-4. The temperature at 5% mass loss (T_5%_) increased from 240 °C for the pristine polymer to 288 °C for the PVA-OP3/TiO_2_-4 sample, while T_30%_ rose from 348 °C to 359 °C. Similarly, the thermal stability index (T_HRI_) improved from 149.4% for the neat polymer to 161.9% for PVA-OP3/TiO_2_-4, confirming the progressive enhancement of heat resistance. These trends can be attributed to the uniform dispersion of TiO_2_ nanoparticles, which act as thermal barriers, slow heat transfer, and promote char formation during pyrolysis. At the highest filler content (PVA-OP3/TiO_2_-5), a slight reduction in T_5%_, T_30%_, and T_HRI_ was observed compared with PVA-OP3/TiO_2_-4, suggesting that excessive TiO_2_ may lead to nanoparticle agglomeration and the formation of localized stress points, consistent with the SEM observation, thereby facilitating degradation. The residual char yield at 700 °C further supports this observation, increasing steadily from 4.5% for the neat polymer to 30.1% for PVA-OP3/TiO_2_-4, followed by a slight decrease to 24.8% for PVA-OP3/TiO_2_-5. For the TiO_2_-containing composites, the TG curves level off near 700 °C, indicating that the main degradation processes have concluded and the residual char yield has stabilized. This behavior demonstrates that moderate TiO_2_ incorporation enhances the thermal stability and char-forming capability of the composite, whereas continuing loading reduces interfacial efficiency and thermal protection. Overall, the optimal TiO_2_ concentration for maximizing the thermal resistance of the PVA-OP3 matrix is identified around the PVA-OP3/TiO_2_-4 formulation. Comparison of the DTG traces (Figure 5b) reveals two dominant degradation features: a major peak centered in the lower-hundreds °C range (primary chain scission/oxidative decomposition of PVA-OP3) and a secondary feature at somewhat higher temperature (further fragmentation/char oxidation). At low to moderate TiO_2_ loadings, the primary DTG peak is slightly attenuated and marginally shifted to higher temperatures relative to the neat polymer, indicating that well-dispersed TiO_2_ nanoparticles act as a physical barrier and hinder heat/mass transfer to the polymer chains, thereby improving thermal stability and reducing the instantaneous rate of weight loss. In contrast, the highest TiO_2_ loading produces a markedly larger and sharper DTG response (greater peak intensity and altered peak shape), together with increased prominence of the secondary degradation feature; this behavior suggests that, beyond an optimal filler concentration, particle aggregation or altered polymer–filler interfacial chemistry can introduce new degradation pathways (or catalytic sites) that accelerate local decomposition.

The heat resistance index (T_HRI_) was determined using the following relationship (Equation (1)) [46]:

(1) $$T_{HRI}=0.49\;\times [T_{5\%}+0.6(T_{30\%}-T_{5\%})]$$

The heat-resistance index (T_HRI_) values are presented in Table 2. PVA-OP3/TiO_2_-0 exhibited a T_HRI_ of 149.4. Notably, the PVA-OP3/TiO_2_-4 sample displayed the highest T_HRI_ among the samples tested. The increased T_HRI_ observed in the PVA-OP3/TiO_2_-4 sample can be attributed to its enhanced thermal stability, which is reflected in a higher amount of carbonaceous residue compared to other samples. This suggests that the higher TiO_2_ content improves the material’s thermal degradation characteristics, leading to greater heat resistance. At higher TiO_2_ content, the material properties are adversely affected. As shown in Table 2, a slight decrease in the thermal stability of the samples can be observed at higher TiO_2_ loadings, accompanied by a corresponding reduction in the char yield measured at 700 °C. The enhanced thermal stability of the PVA-OP3/TiO_2_ composites can be attributed to several synergistic mechanisms. Well-dispersed TiO_2_ nanoparticles act as physical barriers, hindering heat and mass transfer within the polymer matrix and slowing the degradation of polymer chains. Additionally, interfacial interactions between TiO_2_ and PVA segments—likely through hydrogen bonding—restrict segmental mobility, further delaying thermal decomposition. During pyrolysis, the presence of TiO_2_ also promotes the formation of a protective carbonaceous layer, which contributes to higher residual char and improved heat resistance. Together, these effects explain the enhanced T_5_%, T_30_%, T_HRI_, and char yield observed in composites with moderate TiO_2_ content.

### 2.4. Antimicrobial Activity

**Figure 6:**
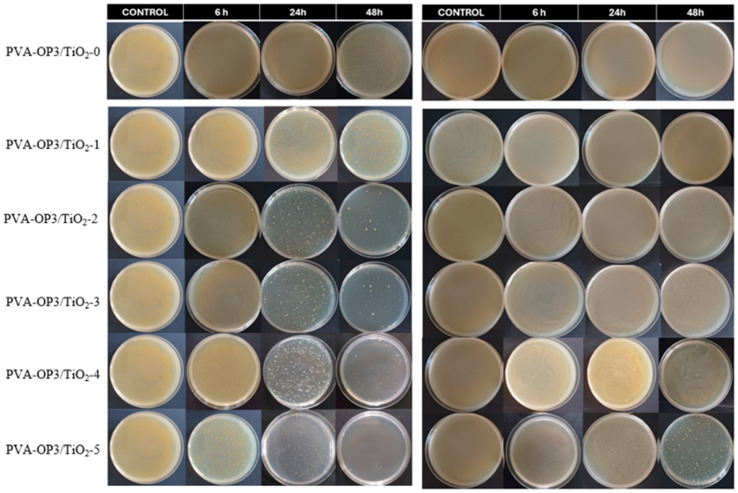
Time-dependent antibacterial activity of PVA-OP3 and PVA–OP3/TiO_2_ composites against *Staphylococcus aureus* and *Escherichia coli*, after 6, 24, and 48 h of incubation. The left panel shows the colony-forming unit of Staphylococcus aureus, and the right panel shows the colony-forming unit of *Escherichia coli*.

The test results showed a progressive increase in antimicrobial activity, correlated with the TiO_2_ concentrations in the PVA-OP3 composites and with the bacterial species tested (Table 3, Figure 6).

For the Gram-positive species (*S. aureus*), the logarithmic reduction increased from moderate values (4.523 log for PVA-OP3/TiO_2_-1) up to an almost complete reduction (7.632 log for PVA-OP3/TiO_2_-5), corresponding to an efficiency of 99.9999% RL (Reduction Log). For the Gram-negative species (*E. coli*), the inhibitory activity was more modest, especially for the samples with lower amounts of TiO_2_ (PVA-OP3/TiO_2_-1, PVA-OP3/TiO_2_-2). The highest logarithmic reduction was obtained for the composite containing the largest amount of TiO_2_ (2.842 log for PVA-OP3/TiO_2_-5), corresponding to an efficiency of approximately 99.856% RL. By comparing the results with those of the sample without TiO_2_ (−0.103 log for PVA-OP3/TiO_2_-0), it can be observed that titanium oxide is responsible for the antimicrobial effect, which becomes more evident as the concentration in the composite increases.

The results of our study indicate that Gram-positive species were more sensitive to TiO_2_ than the Gram-negative species, consistent with some previously published reports [47], although other studies have reported the opposite trend [48,49]. TiO_2_ nanoparticles may exert antimicrobial effects both in darkness, through disruption of membrane integrity and induction of osmotic stress, and under light exposure, which accelerates cell-lysis processes through photocatalysis. Essential components of the cell wall are targeted, such as lipopolysaccharides in Gram-negative bacteria and lipoteichoic acids in Gram-positive bacteria, which can lead to membrane depolarization and impairment of the natural barrier. The presence of carbon quantum dots may also significantly enhance the photocatalytic toxicity [50]. These mechanisms may explain the variable efficiency of TiO_2_ against different types of bacteria.

The variation in sensitivity of the two forms of bacteria is due to the structure of their cell walls. Due to the presence of a thin peptidoglycan layer and a high concentration of lipopolysaccharide in the outer membrane of Gram-negative bacteria, such as *E. coli*, nanoparticles and reactive oxygen species easily penetrate the membranes and cause membrane destabilization. In contrast, Gram-positive bacteria, such as *S. aureus,* have a thicker cell wall composed primarily of peptidoglycan and teichoic acid that initially acts as a physical barrier to the entry of nanoparticles [51]. However, this barrier can eventually become compromised as a result of the accumulation of nanoparticles and the generation of oxidative stress (ROS), ultimately providing the basis for the antimicrobial activity demonstrated in our study.

The antimicrobial efficiency of TiO_2_ nanoparticles ultimately depends on their morphology, crystalline structure, and surface properties, which are determined by the various synthesis methods. Additionally, recent environmentally friendly methods of producing TiO_2_ using plant-derived compounds or microorganisms provide additional flexibility to influence the characteristics of the TiO_2_ nanoparticles so as to maximize the interaction of the nanoparticles with the cell wall of the target bacteria. The ability of TiO_2_ to demonstrate biocidal activity against both Gram-positive and Gram-negative bacteria, including antibiotic-resistant bacteria, is associated with the ability of TiO_2_ to disrupt cell membranes profoundly, inhibit the function of metal ion transport systems, inhibit the function of the respiratory chain, and create dysfunction in the regulation of cellular signaling pathways [51,52].

## 3. Conclusions

The combined morphological, compositional, and thermal analyses clearly demonstrate that the incorporation of TiO_2_ nanoparticles significantly influences both the microstructure and thermal behavior of the PVA-OP3 matrix. SEM observations revealed a transition from a smooth, homogeneous surface in the neat polymer to a progressively rougher and more heterogeneous morphology with increasing TiO_2_ content, reflecting the formation of filler–polymer interfaces and, at higher loadings, the onset of nanoparticle aggregation. These structural features directly correlate with the thermal stability trends observed in the TGA/DTG analyses, where improved dispersion of TiO_2_ (up to PVA-OP3/TiO_2_-4) enhanced heat resistance and char formation through barrier and catalytic stabilization effects. Conversely, excessive TiO_2_ loading promoted agglomeration, diminishing interfacial adhesion and partially reducing the thermal protection efficiency. Overall, these results suggest that controlled incorporation of TiO_2_ nanoparticles—particularly at moderate levels—optimizes filler dispersion, polymer–filler interactions, and ultimately the thermal and structural performance of PVA-OP3-based composites. The antimicrobial performance of the PVA-OP3/TiO_2_ composites is strongly dependent on the TiO_2_ loading and on the structural characteristics of the target microorganisms. The progressive increase in log-reduction values with higher TiO_2_ content confirms the decisive contribution of titania to bacterial inactivation, while the distinct responses of *S. aureus* and *E. coli* highlight the importance of cell-wall architecture in modulating susceptibility. Taken together, the data support that TiO_2_-enhanced PVA systems can provide effective and tunable antimicrobial activity, particularly against Gram-positive bacteria.

## 4. Materials and Methods

### 4.1. Materials

PVA powder (Mw = 30,000–70,000 Da, degree of hydrolysis = 87–90%) was obtained from Sigma-Aldrich (Sigma-Aldrich Chemie GmbH, Eschenstraße 5, 82024 Taufkirchen, Germany). Phenyl dichlorophosphate (purity = 98%) was sourced from TCI EUROPE N.V. (Zwijndrecht, Belgium). Dimethylformamide (DMF) and Titanium dioxide (21 nm) were also acquired from Sigma-Aldrich.

### 4.2. Preparation of the Polymer PVA-OP3

PVA powder (Mw = 30,000–70,000 Da; degree of hydrolysis = 87–90%) supplied by Sigma-Aldrich Chemie GmbH (Taufkirchen, Germany) was used to obtain PVA-OP3 through a controlled nucleophilic substitution reaction. In this process, the hydroxyl (–OH) groups on the PVA backbone react with the chlorine (–Cl) atoms of phenyl dichlorophosphate, following the protocol detailed in our previous publications [12]. The amount of phenyl dichlorophosphate and PVA was calculated to ensure a molar ratio of 1:6 in DMF. The PVA-OP3 polymers were purified through dialysis in distilled water for five days. After purification, the sample was dried under vacuum in an oven at 60 °C [12,14].

### 4.3. Preparation of Composite Films

The PVA-OP3 solution was prepared by dissolving PVA-OP3 powder (1.66 g) in double-distilled water (15 mL) at a concentration of 10% (*w*/*v*). The mixture was stirred at room temperature for 24 h to ensure complete dissolution and uniformity. Subsequently, different amounts of TiO_2_ were added to the PVA-OP3 solution to obtain composite samples designated PVA-OP3/TiO_2_-1 through PVA-OP3/TiO_2_-5. Each mixture was magnetically stirred for an additional 30 min to facilitate homogeneous dispersion. To remove entrapped air, the solutions were left undisturbed at room temperature for 2 h. The degassed mixtures were then cast onto Petri dishes in controlled volumes to form thin films, which were dried under ambient conditions (room temperature) for 72 h before characterization. The detailed composition and processing parameters of the samples are summarized in Table 1.

### 4.4. Methods

#### 4.4.1. FTIR

The chemical structure of the investigated samples was analyzed using a LUMOS Microscope Fourier Transform Infrared (FTIR) spectrophotometer (Bruker Optik GmbH, Ettlingen, Germany) equipped with an attenuated total reflection (ATR) device. Spectra were recorded over a frequency range of 4000–500 cm^−1^ with a resolution of 4 cm^−1^.

#### 4.4.2. SEM

A Verios G4 UC Scanning Electron Microscope (Thermo Scientific, Brno, Czech Republic) was used to examine the surface morphology of the samples. To enhance electrical conductivity and prevent charge buildup during electron beam exposure, the samples were coated with a 6 nm layer of platinum using a Leica EM ACE200 Sputter Coater. SEM analysis was conducted in High Vacuum mode with a secondary electron detector (Everhart-Thornley detector, ETD) at an accelerating voltage of 5 kV.

#### 4.4.3. X-Ray Diffraction Analysis

X-ray diffraction (XRD) analysis was carried out using a Rigaku Miniflex 600 diffractometer equipped with CuKα radiation. Data were collected over a 2θ range of 2° to 50°, with a step size of 0.0025° and a scanning speed of 1° per minute.

#### 4.4.4. TGA

Thermal stability was assessed using a Mettler Toledo TGA-SDTA851e thermogravimetric analyzer (Mettler Toledo, Greifensee, Switzerland) under a nitrogen atmosphere. Measurements were conducted under dynamic conditions with a gas flow rate of 20 mL/min and a heating rate of 10 °C/min, within a temperature range of 25–750 °C. The sample mass varied between 1.6 mg and 2.2 mg. The processing of thermogravimetric curves for the thermal characterization of hybrid materials was carried out using the STAR^e^ software, version 9.1 (Mettler Toledo).

#### 4.4.5. DSC

Differential scanning calorimetry (DSC) analysis was performed using a Mettler Toledo DSC 1 calorimeter (Mettler Toledo, Switzerland). The experiments were conducted under a nitrogen atmosphere with a flow rate of 150 mL/min and a heating rate of 10 °C/min. Samples were placed in aluminum crucibles with perforated lids to facilitate the release of volatile compounds. Samples with masses between 1.8 and 3.7 mg were subjected to two heating processes and one cooling process in the temperature range of 25–200 °C. The evaluation of the obtained DSC curves was performed using the STARe software, version 9.1 (Mettler Toledo). The inflection point was considered to indicate the glass transition temperature.

#### 4.4.6. Antimicrobial Activity

The antimicrobial activity of the samples PVA-OP3/TiO_2_-1, PVA-OP3/TiO_2_-2, PVA-OP3/TiO_2_-3, PVA-OP3/TiO_2_-4, and PVA-OP3/TiO_2_-5 was evaluated using the Time-Kill Assay technique adapted for this type of sample. The antimicrobial potential was tested against the species *Staphylococcus aureus* ATCC 25923 and *Escherichia coli* ATCC 25922. The samples were cut to 1 cm diameter and placed in sterile Eppendorf tubes (2 mL). Afterwards, 2 mL of bacterial suspension (1.5 × 10^8^ CFU/mL) was added and the samples were incubated at 37 °C in a microbiological thermostat (Binder, Roth). The testing times were set at 6, 24, and 48 h of incubation. From the microbial suspensions corresponding to the samples tested at 48 h, decimal dilutions 10^2^–10^8^ were prepared. After incubation, 1 mL of microbial suspension was distributed into sterile Petri dishes (90 mm), Mueller-Hinton nutrient agar (Oxoid, Basingstoke, Hampshire, UK) was added, and after homogenization and solidification, the plates were incubated under the same conditions (24 h/37 °C). The tests were performed in duplicate, and for the obtained values the mean and standard deviation (Mean ± SD) were determined. Finally, the logarithmic reduction (LR) and the percentage of logarithmic reduction (%RL) were calculated in comparison with the culture control (0.5 McFarland turbidity of bacterial suspensions).

## Figures and Tables

**Table 1:** The composition of the investigated films.

Sample Code	PVA-OP3 * (g)	TiO_2_ (g)
PVA-OP3/TiO_2_-0	0.2	-
PVA-OP3/TiO_2_-1	0.2	0.0011
PVA-OP3/TiO_2_-2	0.2	0.0022
PVA-OP3/TiO_2_-3	0.2	0.0115
PVA-OP3/TiO_2_-4	0.2	0.0244
PVA-OP3/TiO_2_-5	0.2	0.0388

* The polymer matrix used as the starting material in this study was prepared in our laboratory, starting from PVA and phenyl dichlorophosphate, following the procedure described in detail in our previous paper [12].

**Table 2:** Thermal stability of the investigated composites.

Samples	*T_g_* (°C)	T_5%_ (°C)	T_30%_ (°C)	T_HRI_ (%)
PVA-OP3/TiO_2_-0	75.33	240	348	149.4
PVA-OP3/TiO_2_-1	79.15	261	350	154.1
PVA-OP3/TiO_2_-2	76.89	274	353	157.5
PVA-OP3/TiO_2_-3	76.99	276	354	158.2
PVA-OP3/TiO_2_-4	77.98	288	359	161.9
PVA-OP3/TiO_2_-5	76.33	284	355	160.0

T_5%_—temperature at which the sample has undergone a 5% weight loss relative to its initial mass during the thermal analysis, T_30%_—temperature at which the sample has undergone a 30% weight loss relative to its initial mass during the thermal analysis, T_HRI_—heat resistance index.

**Table 3:** Log-reduction, CFU viability, and antibacterial efficiency of PVA–OP3/TiO_2_ composites after 48 h incubation.

Sample Code	C	*S. aureus* (UFC × mL^–1^/48 h)			*E. coli* (UFC × mL^–1^/48 h)		
	T0	Mean + SD	LR (Log10) vs. T0	%RL vs. T0	Mean + SD	LR (Log10) vs. T0	%RL vs. T0
PVA-OP3/TiO_2_-0	1.5 × 10^8^	1.9 × 10^8^ ± 2.83 × 10^7^	−0.103	−26.67	1.6 × 10^8^ ± 2.83 × 10^7^	−0.028	−6.67
PVA-OP3/TiO_2_-1	1.5 × 10^8^	4500 ± 198	4.523	99.9970	1.95 × 10^7^ ± 1.06 × 10^7^	0.886	87
PVA-OP3/TiO_2_-2	1.5 × 10^8^	188 ± 29.7	5.902	99.9998	1 × 10^7^ ± 1.41 × 10^5^	1.176	93.33
PVA-OP3/TiO_2_-3	1.5 × 10^8^	229.5 ± 48.8	5.815	99.9998	4.55 × 10^6^ ± 1.91 × 10^6^	1.518	96.97
PVA-OP3/TiO_2_-4	1.5 × 10^8^	109 ± 25.5	6.139	99.9999	2.15 × 10^6^ ± 2.26 × 10^5^	1.844	98.567
PVA-OP3/TiO_2_-5	1.5 × 10^8^	3.5 ± 2.1	7.632	99.9999	2.16 × 10^5^ ± 6.65 × 10^4^	2.842	99.856

## Data Availability

The data that support the findings of the current study are listed within the article.
